# Overview of MitoQ on prevention and management of cardiometabolic diseases: a scoping review

**DOI:** 10.3389/fcvm.2025.1506460

**Published:** 2025-03-11

**Authors:** Mingli Pang, Shidi Wang, Tianyi Shi, Jinsong Chen

**Affiliations:** ^1^ School of Public Affairs, Zhejiang University, Hangzhou, China; ^2^ National Institute for Health Innovation, School of Population Health, The University of Auckland, Auckland, New Zealand; ^3^ Department of Social Medicine and Health Care Management, Fudan University, Shanghai, China; ^4^ Faculty of Medical and Health Sciences, School of Population Health, The University of Auckland, Auckland, New Zealand; ^5^ Faculty of Public Administration, School of Law, Hangzhou City University, Hangzhou, China

**Keywords:** MitoQ, cardiometabolic diseases, metabolic health, cardiovascular diseases, liver health

## Abstract

**Background:**

The exploration of mitochondrial-targeted antioxidants represented a burgeoning field of research with significant implications for cardiometabolic diseases (CMD). The studies reviewed in this scoping analysis collectively highlighted the effect of MitoQ on prevention and management of CMD and underlying mechanisms were discussed, mainly including cardiovascular diseases (CVDs), liver health and others.

**Methods:**

This scoping review aimed to synthesize current research on the health impacts of MitoQ on CMD, focusing primarily on human-based clinical trials. While the primary focus was on human trials, *in vivo* and *in vitro* studies were referenced as supplementary material to provide a broader understanding of MitoQ's mechanisms and potential effects.

**Results:**

This scoping review had synthesized the findings that collectively contributed to the understanding of mitochondrial-targeted antioxidants and their role in CMD.

**Conclusion:**

The synthesis of these findings illustrated a broad spectrum of benefits ranging from enhanced insulin secretion to improved lipid profiles and mitochondrial function, yet the path to clinical application required further investigation on appropriate doses and populations.

## 1 Introduction

Cardiometabolic diseases (CMD) were a group of common but often preventable conditions, including heart attack, stroke, diabetes, insulin resistance and non-alcoholic fatty liver disease (1), representing a significant public health burden globally (2, 3). Cardiovascular diseases (CVDs), liver health and related metabolic disorders were pervasive problems, often linked to mitochondrial dysfunction and oxidative stress (4). MitoQ, a mitochondria-targeted antioxidant, had garnered attention for its potential effects on metabolic health. The role of MitoQ on CMD might have diverse benefits for the prevention and management of CMD (5).

MitoQ was a mitochondria-targeted derivative of Coenzyme Q10 (CoQ10), specifically modified to accumulate within mitochondria more effectively than unmodified CoQ10 (6). The mechanism of action of MitoQ was based on its ability to provide potent antioxidant protection during circulating redox processes while maintaining mitochondrial functional integrity. This property made it a potential agent for the treatment of a wide range of diseases associated with oxidative stress, including cardiovascular diseases, metabolic disorders, neurodegenerative diseases and liver diseases. In detailed, MitoQ reduced mitochondrial oxidative stress, prevented impaired mitochondrial dynamics, and increased mitochondrial turnover by promoting autophagy (mitophagy) and mitochondrial biogenesis, which ultimately contributed to the attenuation of CMD (5), such as fat (7). While MitoQ had been studied in relation to multiple cardiometabolic diseases, the majority of these studies had been conducted at the murine research level rather than in humans.

Recent research emphasized the significance of mitochondrial function in CMD. Previous studies shed light on the role of mitochondria in adipose tissue and the minimal metabolic effects of MitoQ in high-fat-fed mice, respectively (8, 9). These studies indicated that while mitochondrial oxidative stress was intricately linked with metabolic disorders, addressing oxidative stress via mitochondrial targeted antioxidants like MitoQ required deeper exploration. For example, Kim et al. (10) highlighted how obesity exacerbated viral infections in a mouse model, through lipid-induced mitochondrial reactive oxygen species. MitoQ was able to reduce severity of infection and overall mortality, suggesting a broader implication of mitochondrial health in overall metabolic wellness.

CVDs were a significant part of CMD, responsible for an estimated 17.9 million lives annually, which represented 31% of all global deaths (11). These conditions primarily included heart attacks and strokes, often attributed to the accumulation of atherosclerotic plaques in arteries leading to reduced blood flow and oxygen supply (12). Despite advancements in medical therapies, the incidence and impact of CVDs continued to escalate, driven by an aging population and the prevalence of lifestyle-related risk factors such as hypertension, obesity, diabetes, and physical inactivity (13). The pathophysiology of CVD was multifaceted, involving oxidative stress, inflammation, endothelial dysfunction, and altered hemodynamics, necessitating continuous research into novel therapeutic strategies (12).

The liver played a central role in metabolism of lipids and glucose (14); liver disease accounted for two million deaths annually and was responsible for 4% of all deaths (15). Previous studies illustrated that MitoQ could reduce oxidative damage and prevent hepatic fat accumulation (16). MitoQ improved high-fat-induced liver dysfunction by virtue of its antioxidant properties without altering liver fat or mitochondrial bioenergetics (17).

This scoping review would delve into the multifaceted aspects of MitoQ's impact on CMD, especially focusing on metabolic health, CVDs and liver health. By analyzing recent research and data, it aimed to provide a comprehensive overview of the current understanding of mitochondrial-targeted antioxidants in managing CMD, offering insights for future research and potential therapeutic interventions.

## 2 Materials and methods

The methodology for this scoping review on the health impact of MitoQ on CMD was structured to provide a comprehensive overview of the current literature. It adhered to the PRISMA (Preferred Reporting Items for Systematic Reviews and Meta-Analyses) guidelines for scoping reviews. This methodology aimed to identify, assess, and synthesize research findings from various studies to understand the broader implications of MitoQ on CMD.

### 2.1 Identifying the research question

The primary research question guiding this scoping review was: “What was the impact of MitoQ on CMD as evidenced in current scientific literature?” This question sought to explore the effects, both positive and negative, of MitoQ on aspects of CMD, including metabolic health, CVDs and liver health.

### 2.2 Identifying relevant studies

To ensure a comprehensive and relevant synthesis of data, this scoping review adhered to specific criteria for the inclusion and exclusion of studies. These criteria were designed to focus on the most informative and reliable sources of information regarding the cardiometabolic impacts of MitoQ.

### 2.3 Study selection

The inclusion criteria for the studies were:

(1) Published in peer-reviewed journals. Studies specifically discussed MitoQ and its impact on CMD, including metabolic health, CVDs and liver health.
(2) Studies that provided insight into MitoQ and CMD, such as metabolic health (mitochondrial function, oxidative stress, obesity, and diabetes), CVDs (changes in blood pressure, heart rate variability, endothelial function, and other relevant cardiovascular biomarkers) and liver health in the context of MitoQ use.
(3) Studies published in English.
(4) Studies published within the last 15 years.

The exclusion criteria were:

(1) Non-peer-reviewed literature and grey literature.
(2) Studies not relevant to the direct impact of MitoQ on CMD.
(3) Studies not available in full-text.

#### 2.3.1 Charting the data

Data extraction was performed systematically. Key information extracted from each study included the authors, year of publication, study design, sample size, key findings, and conclusions related to the impact of MitoQ on CMD. This process was crucial for synthesizing and comparing results across different studies.

#### 2.3.2 Collating, summarizing, and reporting the results

The collected data were summarized to provide a narrative synthesis of the findings. The synthesis focused on how MitoQ impacted CMD, drawing on specific aspects such as its role in metabolic health, CVDs and liver health.

#### 2.3.3 Consideration of study quality and bias

The quality of the included studies was assessed based on their methodology, sample size, and the robustness of their findings. Potential biases in the studies were identified and noted, particularly biases related to sample selection, experimental design, and reporting.

#### 2.3.4 Consultation exercise

As part of the scoping review, a consultation exercise with experts in the field of mitochondrial research and CMD was conducted. This exercise provided additional insights and helped validate the findings from the literature search.

#### 2.3.5 Ethical considerations

Given that this scoping review only involved the analysis of published literature and did not involve human participants directly, specific ethical approvals were not required. However, all studies included in the review were assessed for their ethical conduct and approval by respective institutional review boards.

### 2.4 Search strategy and sources of data

The search strategy for this scoping review was designed to capture a comprehensive range of studies on the effects of MitoQ on CMD. To achieve a thorough and wide-ranging collection of data, the approach combined traditional database searches with referrals from the MitoQ research team, ensuring a holistic overview of available research.

#### 2.4.1 Online database search

A systematic literature search was conducted across several renowned databases, including PubMed, Scopus, Web of Science, and Google Scholar. This diverse selection of databases was chosen to cover a broad spectrum of scientific publications, from biomedical sciences to clinical research. The search strategy employed a combination of keywords and phrases related to MitoQ (“MitoQ”, “mitochondria-targeted ubiquinol”, “mitoquinone”, “mitoquinol”) and cardiovascular health (“cardiovascular”, “heart disease”, “hypertension”, “endothelial function”), MitoQ and metabolic health (“mitochondrial function”, “oxidative stress”, “obesity”, “diabetes”), MitoQ and liver health (“liver”, “liver diseases”). These terms were used in various configurations and combinations to maximize the retrieval of relevant articles. Boolean operators were utilized to refine the search, and filters such as publication date and language were applied to narrow down the results to the most relevant and recent studies.

#### 2.4.2 Referrals from the MitoQ research team

In addition to the database search, direct referrals from the MitoQ research team were also included as a source of data. This approach was adopted to capture any additional studies that might not be readily available in public databases. The MitoQ research team, being at the forefront of research and development in this area, provided valuable insights and references to studies, including ongoing or recently completed trials, which might not yet be published in academic journals.

#### 2.4.3 Hand-searching and cross-referencing

Following the initial collection of studies, hand-searching was employed as a supplementary method. This involved reviewing the reference lists of key articles to identify any additional studies that might have been missed in the database search. Cross-referencing between the studies obtained from the database search and those referred by the MitoQ research team was conducted to ensure comprehensiveness and avoid duplication. By combining these diverse sources of data, the review aimed to provide a robust and inclusive representation of the current state of research on MitoQ's impact on CMD (including metabolic health, cardiovascular health and liver health). This methodology ensured that the review not only relied on published academic literature but also considered insights and data directly from researchers actively working in this field.

### 2.5 Study selection process

**Figure 1:**
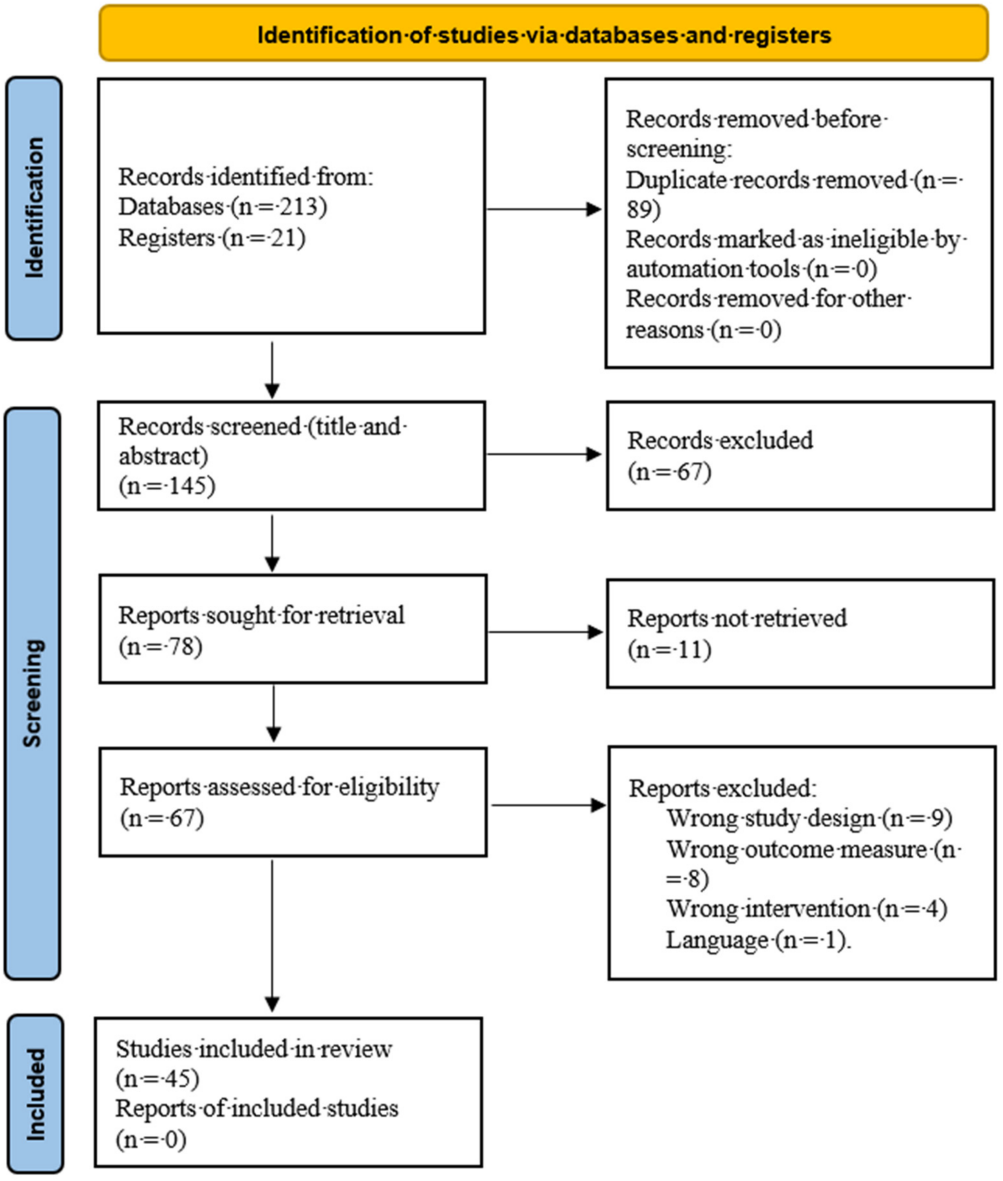
Flowchart PRISMA of study selection.

The process of selecting studies for inclusion in this scoping review was systematic and methodical, aimed at ensuring a comprehensive and unbiased overview of research on MitoQ's impact on CMD, Detailed information about selection process was shown in Figure 1.

Step 1: Initial Screening

The first step involved an initial screening of titles and abstracts retrieved from the database searches and referrals. This preliminary screening was crucial to quickly identify studies that were potentially relevant to the review's objectives.

Two reviewers (MP, SW) independently screened the titles and abstracts. This dual-reviewer approach was employed to minimize the risk of bias and ensure a thorough assessment of each study's relevance.

Step 2: Full-Text Review

Studies that passed the initial screening were then subjected to a full-text review. In this phase, the complete articles were obtained and closely examined to ascertain whether they met the inclusion criteria outlined in the methodology.

Discrepancies in opinions between the two reviewers (MP, SW) about the eligibility of specific studies were resolved through discussion. In cases where consensus could not be reached, a third reviewer (JC) was consulted to make the final decision.

Step 3: Cross-Checking with Referrals

In addition to the database-derived articles, the studies referred by the MitoQ research team were also subjected to the same rigorous selection process. This ensured consistency in the application of the inclusion and exclusion criteria across all sources of data.

Step 4: Documentation and Record Keeping

A record of all decisions made during the study selection process was maintained. This included documenting reasons for excluding studies at the full-text review stage, which was critical for transparency and to allow for a clear understanding of the review process.

Step 5: Final Compilation

The final list of selected studies was compiled after completing the full-text review and resolution of discrepancies. This list represented the studies that were deemed most relevant and suitable for inclusion in the scoping review.

By employing this structured and detailed study selection process, the review aimed to ensure that the final compilation of studies was representative, unbiased, and adhered strictly to the predetermined inclusion and exclusion criteria. This approach was fundamental to the integrity and reliability of the findings presented in the scoping review.

### 2.6 Data extraction and synthesis method

The data extraction and synthesis process for this scoping review was planned and executed to ensure accurate and comprehensive analysis of the selected studies. A standardized data extraction form was developed to gather key information from each study. This form was designed to capture essential details such as study author, year of publication, study design, participant demographics, intervention specifics (including dosage and duration of MitoQ supplementation), and key findings related to CMD. Two reviewers (MP, SW) independently extracted data from each study to minimize the risk of errors and bias. Any discrepancies in the extracted data between reviewers were resolved a third reviewer (JC). The data extraction process was particularly focused on outcomes pertinent to metabolic health (mitochondrial function, oxidative stress, obesity, and diabetes), cardiovascular health (endothelial function, blood pressure measurements, exercise tolerance, and other relevant cardiovascular biomarkers) and liver health.

Given the diversity of study designs and outcomes, a narrative synthesis approach was adopted. This method allowed for a comprehensive and contextual interpretation of the findings, acknowledging the variability and complexity inherent in clinical research. The synthesis involved summarizing the key findings of each study and discussing them in relation to the review's objectives. This included a critical assessment of the strengths and limitations of the studies, as well as an exploration of the implications of the findings for clinical practice and future research. Particular attention was given to variations in study results, seeking to understand and explain discrepancies or consistencies in the context of study methodologies, populations, and MitoQ dosages used.

While the primary focus was on human-based trials, *in vivo* and *in vitro* studies were included as supplementary material. These studies were used to provide additional context and support for the mechanistic understanding of MitoQ's effects on CMD. This supplementary analysis helped in drawing connections between clinical outcomes and underlying biological processes, thereby enriching the review's overall narrative.

The final synthesis was compiled into a coherent narrative, structured to provide a clear and comprehensive overview of the current state of research on the effect of MitoQ on CMD. This synthesis was reported in a manner that emphasizes clarity and accessibility, ensuring that the findings were understandable to both clinical practitioners and researchers in the field. By following this detailed methodology for data extraction and synthesis, the scoping review aimed to provide a thorough and balanced overview of the existing research, highlighting key findings and identifying areas for further investigation in the field of CMD and MitoQ.

## 3 Results

### 3.1 MitoQ and metabolic health

Focusing on metabolic health, a total of thirty studies were analyzed and reviewed, each contributing insights into the role of mitochondrial-targeted antioxidants in metabolic health and cellular function. These studies spanned a diverse range of objectives, from examining the protective effects of antioxidants on pancreatic β-cells to explore the genetic regulation of adiposity and mitochondrial function. The reviewed literature provided a robust foundation for understanding the multifaceted impact of mitochondrial dysfunction and its therapeutic targeting on various aspects of metabolic syndrome and related diseases. A summary table of reviewed studies was shown in Table 1 *Summary Table of Studies on MitoQ's Metabolic Health Impacts*.

**Table 1:** Studies on MitoQ's metabolic health impacts.

#	Reference	Objective	Method	Key findings
1	Lim et al. (28)	Exploring the protection of pancreatic β-cells against oxidative stress and insulin secretion enhancement in glucotoxicity and glucolipotoxicity	Cellular studies on pancreatic β-cells under stress conditions	Showed that mitochondria-targeted antioxidants protect β-cells and improve insulin secretion
2	Mercer et al. (21)	Investigating the effects of MitoQ on metabolic syndrome features in ATM+/–/ApoE–/– mice	Animal study with ATM+/–/ApoE–/– mice, various biochemical assays	Demonstrated that MitoQ decreases features of metabolic syndrome in a mouse model
3	Wang et al. (29)	Studying the impact of Tectorigenin on palmitate-induced endothelial insulin resistance	*in vitro* cellular studies on endothelial cells, analysis of insulin resistance pathways	Found that Tectorigenin attenuates palmitate-induced endothelial insulin resistance by targeting ROS-associated inflammation and IRS-1 pathway
4	Feillet-Coudray et al. (22)	Assessing MitoQ's efficacy in ameliorating metabolic syndrome in an obesogenic diet-fed rat model	Animal study with metabolic assessments	Demonstrated MitoQ's effectiveness in mitigating metabolic syndrome features better than other antioxidants
5	Fink et al. (17)	Investigating the effects of a mitochondrial-targeted Coenzyme Q analog on weight gain and hepatic dysfunction in high-fat-fed mice	Animal study with high-fat diet mice	Observed prevention of weight gain and improvement in hepatic dysfunction with Coenzyme Q analog
6	Fouret et al. (23)	Examining MitoQ's effect on liver mitochondrial cardiolipin in obesogenic diet-fed rats	Animal study with analysis of liver tissues	Found that MitoQ increases liver mitochondrial cardiolipin content in rats on an obesogenic diet
7	Huang et al. (26)	Assessing the effects of Mitoquinone in murine acute pancreatitis	Animal study in a pancreatitis model	Demonstrated that Mitoquinone has protective effects in acute pancreatitis
8	Li et al. (2015)	Studying the contribution of FFA-ROS-P53-mediated mitochondrial apoptosis to bone mass reduction in type 2 diabetes	Cellular and molecular experiments in the context of diabetes	Found that mitochondrial apoptosis via FFA-ROS-P53 pathway reduces osteoblastogenesis and bone mass in diabetes
9	Coudray et al. (2016)	Assessing how a mitochondrial-targeted ubiquinone modifies muscle lipid profile and improves mitochondrial respiration in diet-fed rats	Animal study with diet-induced obese rats	Found improved muscle lipid profile and mitochondrial respiration in obese rats treated with ubiquinone
10	Escribano-Lopez et al. (18)	Investigating the impact of MitoQ on oxidative stress and inflammatory interactions in leukocytes from type 2 diabetic patients	Studying isolated leukocytes with focus on oxidative and inflammatory markers	Showed that MitoQ reduces oxidative stress and improves leukocyte-endothelium interactions in type 2 diabetes
11	Fink et al. (24)	Examining the metabolic effects of a mitochondrial-targeted coenzyme Q analog in obese mice	Animal study with high-fat diet-fed obese mice	Reported significant metabolic effects, including improved insulin sensitivity and reduced fat mass
12	Ju et al. (2017)	Investigating how the antioxidant MMCC ameliorates catch-up growth related metabolic dysfunction	Experimental study on metabolic dysfunction	Discovered that MMCC improves metabolic parameters associated with catch-up growth
13	Escribano-López et al. (19)	Assessing the effects of MitoQ on oxidative stress in pancreatic β cells under hyperglycemic conditions	Cellular experiments under hyperglycemic conditions	Demonstrated that MitoQ alleviates oxidative and endoplasmic reticulum stress, improving mitochondrial function
14	Imai et al. (2018)	Assessing the effect of a mitochondrial-targeted coenzyme Q analog on pancreatic β-cell function in obese mice	Animal study with high-fat-fed obese mice	Demonstrated improved pancreatic β-cell function and energetics in obese mice treated with the coenzyme Q analog
15	Armstrong et al. (30)	Investigating the impact of antioxidants on pancreatic acinar cell bioenergetics and cell fate	Cellular and biochemical assays on pancreatic acinar cells	Found that mitochondrial targeting of antioxidants alters bioenergetics and influences cell fate
16	Bond et al. (9)	Examining the metabolic effects of the antioxidant moiety of MitoQ in adipose tissue of high-fat-fed mice	Animal study with high-fat diet-fed mice, assessment of metabolic parameters	Reported minimal metabolic effects of MitoQ's antioxidant component in adipose tissue of mice
17	Escribano-Lopez et al. (20)	Exploring the role of MitoQ in pancreatic β cell function under hyperglycemia	Cellular experiments measuring mitochondrial function and stress responses	Found that MitoQ alleviates endoplasmic reticulum stress and improves mitochondrial function in β cells under hyperglycemia
18	Marín-Royo et al. (2019)	Investigating mitochondrial oxidative stress in diet-induced obesity in rats	Animal study with biochemical assays	Identified metabolic dysfunction and mitochondrial oxidative stress in diet-induced obese rats
19	Reynolds et al. (2019)	Assessing the impact of age and sex on body composition and glucose sensitivity in C57BL/6J mice	Experimental study using C57BL/6J mice, body composition and glucose sensitivity analysis	Found significant variations in body composition and glucose sensitivity based on age and sex in mice
20	Acín-Pérez et al. (2020)	Exploring the role of Fgr kinase in macrophage activation during diet-induced obesity	Genetic and molecular analysis in a diet-induced obesity model	Identified the necessity of Fgr kinase for proinflammatory macrophage activation in obesity
21	Fink et al. (25)	Examining Mito-Q's effect on neuropathic endpoints in an obese and type 2 diabetic rat model	Animal study with diabetic rats	Found that Mito-Q effectively reduces neuropathy in diabetic and obese rats
22	Walenna et al. (2020)	Studying the effects of Chlamydia pneumoniae infection-induced ER stress on FABP4 secretion in adipocytes	*in vitro* study of adipocyte response to infection	Discovered that infection-induced ER stress leads to FABP4 secretion, implicating a role in metabolic dysfunction
23	Ding et al. (2021)	Investigating the role of peroxisomal β-oxidation in regulating intracellular fatty acids and lipolysis	Experimental study using animal and cellular models, assessment of fatty acid metabolism	Demonstrated that peroxisomal β-oxidation acts as a sensor for intracellular fatty acids and regulates lipolysis
24	Dolezalova et al. (2021)	Studying the impact of accelerated cryoprotectant diffusion kinetics on cryopreservation of pancreatic islets	Experimental study on cryopreservation of pancreatic islets	Demonstrated that enhancing cryoprotectant diffusion improves the efficiency of pancreatic islet cryopreservation
25	Kakimoto et al. (2021)	Investigating the effects of palmitate lipotoxicity on mitochondrial function and redox signaling	Cellular assays on the impact of palmitate on mitochondrial function	Showed that increased glycolysis is an early consequence of palmitate-induced lipotoxicity
26	Chen et al. (49)	Exploring how CD74 ablation rescues cardiac remodeling and dysfunction in type 2 diabetes via pyroptosis and ferroptosis	Cellular and molecular study on diabetic cardiac remodeling	Found that CD74 ablation mitigates cardiac issues in diabetes through influencing cell death pathways
27	Li et al. (2022)	Examining the effects of Mitoquinone on chronic pancreatitis	Experimental study on chronic pancreatitis	Showed that Mitoquinone has anti-fibrotic and antioxidant effects, alleviating chronic pancreatitis
28	Mills et al. (2022)	Investigating how cysteine 253 of UCP1 regulates energy expenditure and adipose tissue inflammation	Biochemical and genetic analysis	Found a link between UCP1 and energy expenditure and adipose tissue inflammation, influenced by sex
29	Moore et al. (31)	Examining the regulation of adiposity by Parkin via coordinating mitophagy with mitochondrial biogenesis in white adipocytes	Genetic and biochemical analysis in adipocytes	Found that Parkin regulates fat accumulation and mitochondrial function in adipocytes
30	Ye et al. (32)	Studying the role of circulating oxidized mitochondrial DNA in chronic inflammation in metabolic syndrome	Analysis of mitochondrial DNA and inflammatory markers	Found that oxidized mitochondrial DNA contributes to inflammation in metabolic syndrome, and MitoQ can alleviate it

#### 3.1.1 Mitochondrial-Targeted antioxidants and metabolic health

A significant portion of the studies within this review focused on how mitochondrial dysfunction influenced metabolic health and the role of mitochondrial-targeted antioxidants, particularly MitoQ, in addressing CMD. Escribano-Lopez et al.'s research on white blood cell function in type 2 diabetes individuals demonstrated the efficacy of MitoQ in reducing oxidative stress, protecting against endoplasmic reticulum stress, and improving mitochondrial function under hyperglycemic conditions. The authors reported MitoQ's amelioration of endoplasmic reticulum stress and NF*κ*B activation, as well as increased insulin secretion and preventing the enhancement of reactive oxygen species (ROS) production and O₂ consumption and decrease in glutathione (GSH) levels that were characteristic under hyperglycemic conditions (18–20). These findings highlighted the potential of MitoQ as a therapeutic agent for metabolic disorders characterized by high blood sugar levels. Similarly, Mercer et al. (21) showed that MitoQ could decrease features (increased adiposity, hypercholesterolemia, and hypertriglyceridemia) of metabolic syndrome in a mouse model, while Feillet-Coudray et al. noted its superiority for xanthine oxidase and NADPH oxidase-dependent ROS production in mitigating metabolic syndrome features in an obesogenic diet-fed rat model (22). This suggested a promising role for MitoQ in managing complex metabolic disorders.

Fouret et al. and Fink et al. presented MitoQ as an intervention that not only improved metabolic syndrome features but also prevented weight gain and hepatic dysfunction, markedly reduced hepatic lipid hydroperoxides and reduced circulating alanine aminotransferase, as well as decreased pathogenic alterations to cardiolipin content and profiles in rodents (17, 23–25). These studies emphasized MitoQ's regulatory capabilities in metabolic pathways, suggesting its utility in managing diet-induced obesity and associated hepatic issues, especially in preventing weight gain. In contrast, Bond et al. reported minimal metabolic effects of MitoQ's antioxidant component in adipose tissue of high fat fed mice, using excised fresh subcutaneous adipose tissue samples from treated mice and measuring their respiratory capacity, indicating that the efficacy of such interventions might be context-dependent and warrant further investigation (9).

#### 3.1.2 Impact on cellular function and disease conditions

The effect of mitochondrial-targeted antioxidants extended beyond metabolic health, influencing various cellular functions in healthy and disease states. For instance, Huang et al. and Li et al. explored the protective effects of MitoQ in acute and chronic pancreatitis models, respectively (26, 27). Moreover, Lim et al. established that mitochondrial-targeted antioxidants could shield pancreatic β-cells from oxidative stress, thereby enhancing insulin secretion and offering a novel approach to managing diabetes-induced β-cell dysfunction (28).

Wang et al. investigated the role of tectorigenin, a mitochondrially active flavonoid, in attenuating palmitate-induced endothelial insulin resistance, shedding light on potential strategies for managing diabetes-associated vascular complications (29). Armstrong et al. found that mitochondrial targeting of antioxidants alters pancreatic acinar cell bioenergetics and influences cell fate, markedly reduced mitochondrial ATP turnover capacity and cellular ATP concentration, suggesting implications for diseases involving acinar cells, like acute pancreatitis (30).

#### 3.1.3 Genetic regulation of metabolic processes

A new frontier in understanding metabolic health was being uncovered by studies investigating genetic regulation. Moore et al. provided a mechanistic insight, showing that Parkin regulated adiposity by coordinating mitophagy with mitochondrial biogenesis in white adipocytes (31). This study underscored the importance of mitochondrial dynamics in adipose tissue function and the pathophysiology of obesity.

Additionally, Ye et al. delved into the role of circulating oxidized mitochondrial DNA in chronic inflammation in metabolic syndrome (32). Their discovery that MitoQ could alleviate DNA-induced inflammation opens avenues for addressing systemic inflammation in CMD. This finding pointed to the potential of targeting mitochondrial integrity and function to manage systemic inflammatory responses that were central to metabolic syndrome.

These studies collectively underscored the promising roles of mitochondrial-targeted interventions in metabolic health. They highlighted the therapeutic promise of mitochondrial antioxidants in ameliorating various aspects of metabolic dysfunction, from improving insulin secretion and reducing weight gain to manage systemic inflammation. However, some contradictory findings also indicated the need for further research to elucidate the differential effects in various metabolic contexts and to optimize therapeutic strategies for clinical use. The results affirmed the importance of a nuanced approach to treatment, considering individual genetic and metabolic profiles to maximize therapeutic efficacy.

### 3.2 Mitoq and CVDs

Focusing on CVDs, the scoping review analyzed six primary studies, each offering insights into the cardiovascular impacts of MitoQ supplementation. These studies provided a broad perspective on how MitoQ might benefit various aspects of cardiovascular health across different populations. *in vivo* studies showed that MitoQ had demonstrated multiple positive effects on cardiovascular health (33, 34), anti-oxidative stress (34, 35) and exercise tolerance (33, 34, 36, 37), especially in patients with chronic conditions. Different studies had produced different conclusions about the effects of muscle recovery, with some not finding significant benefits (38). A summary table of reviewed studies was illustrated in Table 2 *Studies on MitoQ's Cardiovascular Health Impacts*.

**Table 2:** Studies on mitoQ's cardiovascular health impacts.

#	Reference	Objective	Method	Key Findings
1	Park et al., (36)	Examining the roles of vascular mitochondria in endothelial function, arterial stiffness, exercise tolerance, and skeletal muscle function in patients with PAD	Randomized controlled trial and the intervention of acute MitoQ intake	Improved endothelial function, antioxidant enzyme activity, and exercise tolerance
2	Tharpe et al., (35)	Assessing central hemodynamics, arterial stiffness, and measures of oxidative stress in healthy young adults before and after acute MitoQ (or placebo) supplementation	Abstract review and the intervention of MitoQ supplementation	Assessed central hemodynamics, arterial stiffness, and oxidative stress
3	Broome et al., (38)	Examining the effect of mitochondria-targeted antioxidant supplementation on recovery of muscle function following exercise	Randomized clinical Trial and the intervention of MitoQ supplementation	No significant effect on muscle soreness or recovery post-exercise
4	Masoumi-Ardakani et al., (33)	Investigating the effects of mitochondrial-targeted antioxidants (mitoAOXs) on glycaemic control, cardiovascular health, and oxidative stress outcomes in humans	Randomized clinical Trial and the intervention of Endurance Training and MitoQ	Improved cardiovascular function, oxidative stress, and inflammation
5	Masoumi-Ardakani et al., (34) (second study)	Evaluating the effects of MitoQ supplementation and moderate endurance training, alone and in combination, on cardiac function, blood pressure, the circulatory levels of miRNA-21 and miRNA-222, and oxidative status in individuals with hypertension	Randomized double-blind clinical trial and the intervention of MitoQ and moderate endurance training	Improved cardiovascular function, highlighting miR-21 and miR-222
6	Kirkman et al., (37)	Investigate the effects of a mitochondria-targeted ubiquinol (MitoQ) on vascular function and exercise capacity in chronic kidney disease	Randomized controlled pilot study and the intervention of MitoQ ubiquinol supplementation	Impacted vascular function and exercise capacity in chronic kidney disease

The comparative analysis of the reviewed studies revealed several key observations and implications regarding MitoQ's role in CVDs. Firstly, the diversity in the study populations was noteworthy, ranging from patients with specific conditions, such as individuals with hypertension (33), peripheral artery disease (36) and chronic kidney disease (37), to healthy young adults (35). The wide-ranging demographic representation provided valuable insights into the effects of MitoQ across various groups, suggesting its potential relevance to a broad spectrum of individuals. Secondly, there was notable variation in the interventions across these studies, particularly concerning the dosage and duration of MitoQ supplementation, some studies, such as Masoumi-Ardakani et al., combined MitoQ with endurance training, revealing enhanced cardiovascular outcomes and reductions in oxidative stress and inflammation (33, 34), while others focused on standalone supplementation, which didn't observe significant effects on post-exercise recovery (38).

While majority of the studies reported positive effects of MitoQ on cardiovascular parameters, such as improved endothelial function (36), reduced oxidative stress and better blood pressure regulation (33, 34), a few studies, for example, Broome et al. didn't observe significant effects of MitoQ on physiological outcomes such as inflammatory recovery post-exercise (38), highlighting the complexity of MitoQ's impact on different health aspects.

### 3.3 MitoQ and liver health

Focusing on liver health, a total of 9 articles were reviewed in this study, these studies contributed insights into the potential benefits of MitoQ in liver health management. *in vivo* studies showed that in animal models (e.g., mice or rats) the role of MitoQ in specific diseases or pathological states was analysed, mainly related to liver function, steatosis, fibrosis, and inflammation (16, 39–45). The only *in vitro* study, using hepatocytes and neuronal cell lines, revealed the cytotoxicity of MitoQ at high concentrations while comparing the toxicity and efficacy of different mitochondria-targeted antioxidants (46). A summary table of the reviewed articles on MitoQ's impacts on liver health was presented in Table 3 *Studies on MitoQ's Liver Health Impacts*.

**Table 3:** Studies on mitoQ's liver health impacts.

#	Reference	Objective	Method	Key findings
1	Gerard Li et al. (2019)	Examining the impact of maternal cigarette smoke exposure on offspring's metabolic profile and hepatic damage, and whether maternal MitoQ supplementation during gestation can affect these changes.	A mouse model where female Balb/c mice were either exposed to air or smoke exposure.	Maternal MitoQ supplementation reduced hepatic mitochondrial oxidative stress and improved markers of mitophagy and mitochondrial biogenesis
2	Fernandes et al. (46)	Evaluate the effects of MitoQ and other mitochondria-targeted and untargeted antioxidants on human neuronal and hepatic cell lines.	Caucasian hepatocyte carcinoma (HepG2) and differentiated human neuroblastoma (SH-SY5Y) cells were treated with increasing concentrations of the different molecules for a period of 48 h.	MitoCINs derivatives reduced cell viability at concentrations about six times higher than those observed with MitoQ and SkQ1. MC4 and MC7.2 displayed around 100–1,000 times less cytotoxicity than SkQ1 and MitoQ.
3	Wan et al. (40)	Investigating the role of NDUFS1 in the pathogenesis of PNALD and its underlying mechanism.	Hepatic proteomics analysis of PNALD patients and a PNALD rat model.	The downregulation of NDUFS1 expression increases oxidative stress, contributing to the progression of PNALD, furtherly, treatment with MitoQ or overexpression of NDUFS1 can alleviate PNALD by reducing oxidative stress.
4	Fink et al. (2021)	Investigating the effect of mitoquinone (mitoQ) on liver function in rats treated with high-fat (HF) to induce obesity and in rats treated with HF plus streptozotocin (STZ) to model a severe form of type 2 diabetes.	Measuring body weight and food consumption, assessing hepatic fat content and lipid hydroperoxides, and examining circulating markers of liver function and oxidative stress.	MitoQ significantly improved glycemia in HF rats but not in the diabetic rats. MitoQ also altered several hepatic metabolic pathways in HF-fed obese rats toward those observed in control normal chow-fed non-obese rats.
5	Turkseven et al. (41)	Evaluating if the anti-inflammatory activity of acetylsalicylic acid (ASA) and the mitochondria-targeted antioxidant effect of mitoquinone could hinder the progression of non-alcoholic steatohepatitis.	Two experimental groups were then treated orally with ASA or MitoQ.	Both ASA and MitoQ can reduce steatosis and inflammation in mice with NASH. It suggests that the beneficial effects of these treatments are associated with a decrease in oxidative stress.
6	Turkseven et al. (42)	Investigating the effect of mitochondria-targeted antioxidant mitoquinone in an experimental model of cirrhosis.	Mitoquinone or vehicle was administered from 3rd to 28th day after common bile duct ligation.	Mitoquinone treatment significantly reduced liver inflammation and fibrosis in cirrhotic rats and also decreased the expression of several genes associated with inflammation and fibrosis.
7	Chacko et al. (43)	Investigating the effects of mitochondria-targeted ubiquinone (MitoQ) (5 and 25 mg/kg/day for 4 weeks) in male Sprague-Dawley rats consuming ethanol using the Lieber-DeCarli diet with pair-fed controls.	Lieber-DeCarli diet with pair-fed controls.	MitoQ had a minor effect on the ethanol-dependent decrease in mitochondrial respiratory chain proteins and their activities; however, it did decrease hepatic steatosis in ethanol-consuming animals and prevented the ethanol-induced formation of 3-NT and 4-HNE.
8	Hao et al. (44)	Investigating if MitoQ could preserve mitochondrial ALDH2 activity and speed up acetaldehyde clearance, thereby protects against alcoholic liver disease.	Pair-fed.	Alcohol-induced ER stress and apoptotic cell death signaling were reversed by MitoQ.
9	Mukhopadhyay et al. (45)	Exploring the spatial-temporal relationship of oxidative/nitrative stress and inflammatory response during the course of hepatic I/R and the possible therapeutic potential of mitochondrial-targeted antioxidants.	A mouse model of segmental hepatic ischemia-reperfusion injury.	Mitochondrially targeted antioxidants, MitoQ or Mito-CP, dose-dependently attenuated I/R-induced liver dysfunction, the early and delayed oxidative and nitrative stress response, and mitochondrial and histopathological injury/dysfunction, as well as delayed inflammatory cell infiltration and cell death.

Multiple studies had shown that MitoQ reduced oxidative stress in the liver and improved liver cell survival by reducing mitochondrial oxidative stress levels (45). Studies also had found that MitoQ could help improve mitochondrial function, including promoting mitochondrial autophagy and mitochondrial biosynthesis, leading to improved liver health (39, 44). Furtherly, MitoQ had potential therapeutic effects on specific liver diseases, such as non-alcoholic fatty liver disease, liver fibrosis, and hepatitis, by reducing the severity of the disease and improving the associated pathophysiologic processes (42–44).

## 4 Discussion

This scoping review had synthesized the findings from 45 studies that collectively contributed to the understanding of mitochondrial-targeted antioxidants and their role in CMD. The evidence suggested promising therapeutic potential for compounds such as MitoQ in mitigating various metabolic and cellular dysfunctions.

The discussion of mitochondrial function in CMD was complex, the study by Moore et al. presented Parkin as a regulator of adiposity, indicating a key role for mitochondrial quality control in adipocyte metabolism (31). However, the intricacies of mitophagy and its regulation by Parkin in human adipocytes remained to be fully elucidated.

Recent research had explored the potential role of MitoQ in managing CMD. Mitochondrial dysfunction and oxidative stress were implicated in the pathogenesis of conditions such as CVDs, diabetes, liver diseases and metabolic syndrome. MitoQ, by selectively targeting mitochondria and reducing reactive oxygen species production, had shown promise in preclinical studies for its ability to improve mitochondrial function and attenuate oxidative stress. For instance, studies had demonstrated that MitoQ supplementation can enhance endothelial function (47), and reduce oxidative damage to lipids and proteins (48). These studies underscored the importance of mitochondrial health and oxidative balance in the pathophysiology of CMD. Moreover, studies like those by Escribano-Lopez et al. and Chen et al. highlighted the role of mitochondrial-targeted antioxidants in reducing oxidative stress and inflammation (18–20, 49). However, the complexity of redox biology in human physiology suggested that the balance between pro-oxidant and antioxidant mechanisms might be more delicate than currently understood. Oversimplification of these dynamics could introduce bias in interpreting the efficacy of antioxidant therapies (36, 38).

In CVDs, the effect of MitoQ mainly existed in serving as cardiovascular health protectors and chronic disease management tools, as well as enhancing healthy individual function. MitoQ enhanced endothelial function by improving vascular mitochondrial function (36), while significantly reducing arterial stiffness, improving hemodynamic parameters, and attenuating atherosclerosis (35). Furtherly, in patients with chronic kidney disease, MitoQ helped to enhance vascular elasticity and exercise tolerance (37), another study also showed MitoQ increased endothelial function in non-exercisers with lower cardiorespiratory fitness (50). Some studies had illustrated that MitoQ didn't significantly improve recovery of muscle function or reduce muscle soreness after exercise (38), while other study clarified that training-induced increased in peak power were enhanced following MitoQ supplementation (51).Furthermore, when combined with endurance training, it was more effective in improving cardiovascular and antioxidant function (33, 34). Although MitoQ had a limited effect on post-exercise recovery, it had potential benefits in optimizing vascular function and antioxidant status, as well as reducing cardiac hypertrophy (47, 52).

For liver health, MitoQ targeted mitochondria and significantly reduced oxidative stress, alleviated liver inflammation and fat accumulation, inhibited liver fibrosis, and improved metabolic abnormalities, it demonstrated protective effects in various liver disease models (16, 39–46). MitoQ could protect the liver from some pathways; some studies focused on chronic alcohol-induced liver disease study, illustrated the pathway from reducing alcohol-induced hepatic fat deposition, preventing the formation of ethanol-induced oxidation products (43), as well as ameliorating endoplasmic reticulum stress and apoptotic signalling induced by alcohol (44), furtherly, some studies focused on other types liver disease illustrated that it could alleviate liver fibrosis (42, 53) as well as relieve arsenic-induced acute liver injury and immune imbalance (54, 55).

However, some studies, such as the one conducted by Broome et al. (38), did not find significant effects of MitoQ in muscle recovery, which contrasted with the positive outcomes reported in other research. However, they did report decreased inflammation via F2-isoprostanes after exercise. This discrepancy highlighted the need for caution in interpreting the results and suggested that further research was necessary to understand the conditions under which MitoQ was most effective.

This scoping review provided valuable insights into the therapeutic potential of MitoQ for the prevention and management of CMD, however, several limitations should be acknowledged. Firstly, the variability in study designs and methodologies across the included, differences in study populations ranged from healthy individuals to patients with various conditions, as well as inconsistencies in dosage and duration of MitoQ supplementation, make it challenging to generalize findings and draw definitive conclusions. Secondly, most of the evidence reviewed originates from preclinical studies and small-scale clinical trials, lack of the robust, large-scale clinical trials to validate the efficacy and safety of MitoQ in diverse patient populations. Thirdly, there was a lack of consistency regarding the dosage and duration of MitoQ supplementation in the reviewed studies, this variability complicated the interpretation of findings, given the limited clinical trials, caution should be exercised when considering the use of this drug.

For metabolic diseases, the findings from this review underscored the potential of mitochondrial-targeted therapies in treating metabolic disorders, such as improved insulin sensitivity and reduced inflammation. For CVDs, MitoQ's ability to enhance endothelial function and mitigate oxidative damage could play a significant role in the prevention and management of CVDs. For liver health, recent studies had noted the potential of MitoQ in promoting liver health. Evidence suggested that MitoQ could effectively reduce oxidative damage and prevent hepatic fat accumulation, thereby mitigating liver dysfunction, particularly in cases induced by high-fat diets.

## 5 Conclusion

The exploration of mitochondrial-targeted antioxidants represented a burgeoning field of research with significant implications for CMD. The studies reviewed in this scoping analysis collectively highlighted the therapeutic potential of compounds such as MitoQ in modulating various aspects of CMD, including CVDs, liver health and metabolic health. The synthesis of these findings illustrated a broad spectrum of benefits ranging from enhanced insulin secretion to improved lipid profiles and mitochondrial function.

The potential of mitochondrial antioxidants to serve as a novel therapeutic approach for CMD was clear, yet the path to clinical application was paved with the need for further investigation. Future research need not only focus on confirming these findings in human populations but also on understanding the precise mechanisms by which these compounds exert their effects. This would entail detailed studies into mitochondrial dynamics, the interplay between different cellular stress pathways, and the impact of genetic and environmental factors on individual responses to treatment.

## Data Availability

The original contributions presented in the study are included in the article/Supplementary Material, further inquiries can be directed to the corresponding author.
